# Characterisation of the *Theileria orientalis* Piroplasm Proteome across Three Common Genotypes

**DOI:** 10.3390/pathogens11101135

**Published:** 2022-09-30

**Authors:** Cheryl Jenkins, Melinda L. Micallef, Matthew P. Padula, Daniel R. Bogema

**Affiliations:** 1 NSW Department of Primary Industries, Elizabeth Macarthur Agricultural Institute, Menangle, NSW 2568, Australia; 2 School of Life Sciences, Faculty of Sciences, University of Technology, Sydney, NSW 2007, Australia

**Keywords:** *Theileria orientalis*, piroplasm, proteomics, TX-114, membrane protein, erythrocyte, metabolism, haemoglobin

## Abstract

*Theileria orientalis* is an emerging apicomplexan pathogen of cattle occurring in areas populated by the principal vector tick, *Haemaphysalis longicornis*. Unlike transforming *Theileria* spp. that induce cancer-like proliferation of lymphocytes via their schizont stage, *T. orientalis* destroys host erythrocytes during its piroplasm phase resulting in anaemia. The underlying pathogenic processes of *T. orientalis* infection are poorly understood; consequently, there are no vaccines for prevention of *T. orientalis* infection and chemotherapeutic options are limited. To identify antigens expressed during the piroplasm phase of *T. orientalis*, including those which may be useful targets for future therapeutic development, we examined the proteome across three common genotypes of the parasite (Ikeda, Chitose and Buffeli) using preparations of piroplasms purified from bovine blood. A combination of Triton X-114 extraction, one-dimensional electrophoresis and LC-MS/MS identified a total of 1113 proteins across all genotypes, with less than 3% of these representing host-derived proteins. Just over three quarters of *T. orientalis* proteins (78%) identified were from the aqueous phase of the TX-114 extraction representing cytosolic proteins, with the remaining 22% from the detergent phase, representing membrane-associated proteins. All enzymes involved in glycolysis were expressed, suggesting that this is the major metabolic pathway used during the *T. orientalis* piroplasm phase. Proteins involved in binding and breakdown of haemoglobin were also identified, suggesting that *T. orientalis* uses haemoglobin as a source of amino acids. A number of proteins involved in host cell interaction were also identified which may be suitable targets for the development of chemotherapeutics or vaccines.

## 1. Introduction

The haemoprotozoan *Theileria orientalis* is an apicomplexan parasite with life stages that cycle between ticks and cattle. Cattle infected with high burdens of the Ikeda genotype often become anaemic with clinical signs including tachypnoea, lethargy, ataxia, abortion in pregnant animals and mortality in up to 10% of cases [1,2]. Stressors such as parturition, lactation and transport of infected animals exacerbate disease [1,2]. The geographical range of *T. orientalis* Ikeda has expanded significantly in the past two decades facilitated by the widespread occurrence of its principal tick vector, *Haemaphysalis longicornis*. Clinical disease outbreaks caused by *T. orientalis* Ikeda (formerly referred to as *Theileria sergenti*) have been reported in Japan and Korea for many years [3,4]; however recent incursions of *T. orientalis* Ikeda have been reported in the Asia-Pacific region [5,6,7,8] and the eastern United States [9].

Measures for control of this parasite are limited. In Australia and New Zealand, the parasite is now endemic across the range of the vector tick with widespread immunity observed in adult cattle [10,11,12]. However, mixing of naïve and endemic animals remains a major risk for clinical outbreaks of disease and passive immunity confers little protection on young calves, with high parasitaemias routinely observed between 3 and 12 weeks of age [13]. Control of vector ticks via acaricides is recommended as treatments and chemotherapeutics for *T. orientalis* infection are currently limited or unavailable. Buparvaquone, which is used for the treatment of *Theileria parva* infection (East Coast fever) in Africa, is also effective against *T. orientalis* if used during the earlier stages of infection; however, it is not approved for use in some countries and has a lengthy withholding period due to the retention of residues in meat and milk products [14]. Blood transfusions are sometimes used to treat anaemia due to *T. orientalis* infection; however, this process is costly and is not always successful [11].

While the “transforming” theilerias (such as *Theileria parva* and *Theileria annulata*) induce uncontrolled proliferation of B and T lymphocytes and/or macrophages via their schizont phase, it is the intraerythrocytic piroplasm phase of *T. orientalis* that is responsible for the observed pathology. In this respect, *T. orientalis* is more akin to *Babesia* spp. and haemosporidians such as *Plasmodium* spp. which cause destruction or sequestration of erythrocytes [15]. Nonetheless, the processes by which pathogenesis occur in this organism are poorly understood, hampering the development of chemotherapeutics or prophylactic measures such as vaccines. In this study, we conducted a proteomic analysis of *T. orientalis* piroplasms to identify expressed proteins that may form the basis of future treatment modalities.

## 2. Materials and Methods

### 2.1. Collection and Propagation of T. orientalis Strains

*T. orientalis* strains were sourced from Australian cattle testing PCR positive for a single major piroplasm surface protein (MPSP) genotype (Ikeda, Chitose or Buffeli) [5,16,17] and have been described in detail previously [18]. The strains are named according to their location of isolation: the Robertson strain was isolated from New South Wales is of the Ikeda genotype; the Fish Creek and Goon Nure strains were isolated from Victoria and are of the Chitose and Buffeli genotypes respectively. The strains were propagated in splenectomised calves inoculated with stabilates of each isolate at the Tick Fever Centre (Wacol, QLD, Australia) as previously described [18]. Details of blood testing and collection and the purification of parasites using the nitrogen cavitation method followed by differential centrifugation are also as previously described [18,19]. Purified piroplasm preparations were maintained at −80 °C until required for proteomic analysis.

### 2.2. Proteomics

#### 2.2.1. Protein Purification

Proteins were purified from *T. orientalis* Ikeda, Chitose and Buffeli piroplasms using an in-house Triton X-114 method to separate aqueous and detergent soluble proteins. Approximately 0.1 g of piroplasms were thawed on ice and resuspended in 1 mL cold 1% triton buffer (1% TX-114, 10 mM Tris pH 8.0, 150 mM NaCl, 1 mM EDTA). Samples were extracted overnight on a rotary shaker at 4 °C. Samples were centrifuged cold at 13,000 rpm for 15 min after which the supernatant was placed in a new tube and incubated for 10 min at 37 °C. The pellet represented TX-114 insoluble pellet. The supernatant was phase separated by centrifuging for 5 min at 10,000 rpm. The top phase (representing the aqueous phase) was pipetted into a new tube and 20 μL TX-114 was added. A 1 mL aliquot of 1% Triton buffer was added to the detergent phase and the samples were re-extracted on a rotary shaker as described above, but for 4 h. The phases were separated again with a 10 min incubation at 37 °C followed by centrifugation at 10,000 rpm for 5 min. The top and bottom phases (aqueous and detergent respectively) were separated into 2 × 50 mL centrifuge tubes and each sample was mixed with 10 volumes of ice-cold acetone to precipitate proteins. After an overnight incubation at −20 °C, precipitated proteins were pelleted by spinning at 13,000 rpm and 4 °C for 30 min. The acetone was decanted off each sample and the pellets air dried for a period of 30 min. The pellet from the aqueous phase was resuspended in 0.5 mL of SSS buffer (8 M Urea, 100 mM DTT, 4% chaps, 0.8% ampholytes, 40 mM Tris) and the pellet from the detergent phase was resuspended in 0.25 mL MSS buffer (5 M urea, 65 mM DTT, 2% chaps, 0.8% ampholytes, 40 mM tris, 2 M thiourea, 2% sulfobetaine). After 30 min incubation at RT with alternate cycles (×4) of sonication (30 s) and vortexing (15 s), samples were centrifuged at 13,000 rpm for 15 min. Supernatants were stored at −20 °C in fresh tubes.

The aqueous and detergent-phase proteins were passed through a 300 kDa filter (Pall Life Sciences, Port Washington, NY, USA) and were then desalted using Micro BioSpin P6 gel columns (BioRad Laboratories, Hercules, CA, USA). Aliquots of each fraction were analysed by electrophoresis on Criterion 12% TGX precast gels (BioRad Laboratories) at 200 V for 1 h. For visualisation of protein bands, gels were washed 3 × 10 min with MilliQ water and then stained with GelCode Blue stain reagent (ThermoFisher Scientific, Waltham, MA, USA). Destaining was achieved with MilliQ water.

#### 2.2.2. Trypsin In-Gel Digestion “Slice and Dice” Protocol

Gel slices were taken from each of the stained gels with a scalpel blade, were diced into 1 mm^2^ cubes and kept moist in individual 0.6 mL microfuge tubes with deionised water. Excess water was removed, and the gel pieces were equilibrated with bicarbonate solution (100 mM NH_4_HCO_3_). A 200 µL volume of 50% acetonitrile-50 mM NH_4_HCO_3_ was added to each tube to destain the gel; samples were vortexed and incubated for 10 min. To dehydrate the samples, excess liquid was removed, 200 µL of 100% acetonitrile was added, and samples were vortexed and incubated at room temperature for 10 min.

To reduce and alkylate the proteins, the gel pieces were rehydrated with 50 µL of 5 mM tributylphosphine/20 mM acrylamide in NH_4_HCO_3_ and incubated for 90 min. Gel pieces were then washed with 100 mM NH_4_HCO_3_ for 5 min, followed by 50% acetonitrile-50 mM NH_4_HCO_3_ for 5 min and finally were dehydrated with 200 µL of acetonitrile. Following dehydration, any remaining liquid was removed and to generate peptides, the gel pieces were rehydrated with 12.5 ng/µL of trypsin in 100 mM NH_4_HCO_3_ at 4 °C for 30 min. Following re-swelling of the gel, NH_4_HCO_3_ was added as required to keep the gel covered with liquid. Protein digestion was achieved overnight at 37 °C.

#### 2.2.3. Mass Spectrometry

Following trypsin digestion, the samples were sonicated in a waterbath for 10 min to extract the peptides and centrifuged to release the supernatant. The resulting digest solution was transferred to a clean tube. A 50% acetonitrile-2% formic acid solution (30 µL) was added to the gel pieces with sonication and centrifugation repeated as above. The supernatant was then added to the previous supernatant sample. This step was repeated again yielding a peptide extract volume of >60 µL. Concentration of the samples to a volume of 15 µL was achieved using a SpeedVac vacuum concentrator. Prior to analysis, microparticulates were removed by centrifuging the samples at 14,000 rpm for 10 min. The samples were then loaded into an autosampler tube and into a QSTAR Elite Liquid chromatography-tandem mass spectrometry (LC-MS/MS) system for analysis [20]. MS/MS MASCOT searches were performed against a composite database consisting of the Bos taurus proteome and the known sequences of apicomplexan parasite proteins to overcome any omissions in the available *T. orientalis* protein sequences. Scores were assigned to protein matches and peptide matches with an E-value of <0.05 were considered statistically significant and the peptide identification deemed valid. The relative abundance of proteins was calculated using the exponentially modified protein abundance index (emPAI) [21] within MASCOT. Duplicate matches were removed from the final numbers.

## 3. Results and Discussion

### 3.1. Electrophoretic Analysis of Purified T. orientalis Proteins

Aqueous and detergent soluble *T. orientalis* proteins were electrophoresed on 1D SDS-PAGE gels (Criterion 12% TGX precast gels, BioRad). The aqueous and detergent fractions for each *T. orientalis* genotype are shown in Figure S1A,B. respectively.

### 3.2. LC MS/MS Identification of Purified T. orientalis Proteins

Eight, eleven and twelve individual gel slices were analysed from the aqueous phase of Chitose, Ikeda and Buffeli genotype piroplasms respectively. When examining the detergent phase, twelve slices for Chitose and eleven for both Ikeda and Buffeli were analysed (highlighted in Figure S1). All samples were analysed using liquid chromatography-tandem mass spectrometry (LC-MS/MS). Spectra for individual gel slices were then converted into a peak list and Mascot MS/MS searches were performed. A total of 1113 proteins were identified across all *T. orientalis* genotypes. Of these proteins only 2.8% (*n* = 31) represented *Bos taurus* proteins and a further 1.9% (*n* = 21) represented other contaminating proteins, indicating efficient purification of *T. orientalis* piroplasms from bovine blood.

A total of 1061 target proteins (95.3%) were identified that matched either the reference *T. orientalis* Shintoku genome or related apicomplexan parasites and other protozoans. This represents approximately one quarter of the total predicted proteome of *T. orientalis* with 4058, 3980 and 3924 genes predicted in the Ikeda, Chitose and Buffeli genomes respectively [22,23]. Approximately three quarters (78%; *n* = 829) of the proteins identified were aqueous phase proteins, while 22% (*n* = 232) were from the detergent phase of the TX-114 extraction, representing membrane-associated and other hydrophobic proteins. This proportion of detergent phase proteins was similar to those recovered from *Theileria annulata* schizonts using TX114 [24]. The total number of proteins recovered from the *T. orientalis* Ikeda and Buffeli genotypes was greater than for the Chitose genotype (Table 1). Indeed, one limitation of this study was that the number of proteins identified per genotype was not sufficient to allow for meaningful cross-comparison of all proteins expressed between pathogenic and apathogenic types. The inability to culture *T. orientalis* in vitro under standard conditions, with experimental infection of animals used for propagation, may also have influenced the protein profile across genotypes.

### 3.3. Protein Functional Groups and Relative Abundance

**Figure 1:**
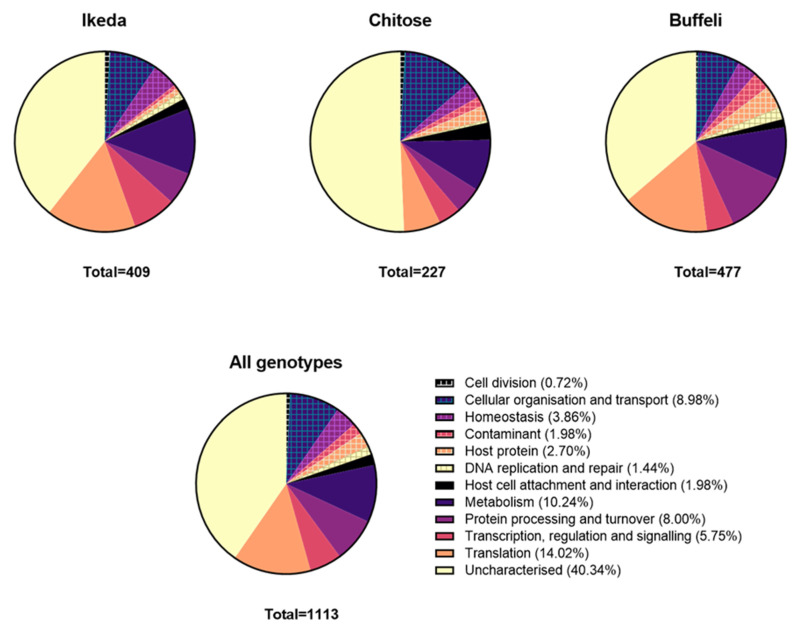
Proportion of *T. orientalis* proteins identified by functional group.

Across all genotypes, approximately 40% of the proteins identified were yet uncharacterised proteins with unknown functions; however putative functions could be assigned to the remaining 60% of proteins. Where putative functions could be assigned, the most common functional designations were metabolism, translation, transcription, regulation and signalling, protein processing and turnover and cellular organisation and transport (Figure 1 and Table S1). As expected, proteins with cytosolic functions, such as those involved in translation or metabolism, were generally identified in the aqueous fractions; while membrane-associated proteins, such as transporters and major surface proteins were located in the detergent fractions. The detergent phases also contained a high proportion of uncharacterised proteins (Table S2).

The relative abundance of proteins as estimated via emPAI indicated similarities between the most abundant proteins across *T. orientalis* genotypes. In the aqueous phase fractions, proteins involved in translation, such as elongation factor Tu and elongation factor 1α and ribosomal proteins, proteins involved in homeostasis such as heat shock proteins and redox proteins, metabolic enzymes and cytoskeletal proteins such as actin and tubulin were amongst the most abundant. A number of proteins were also identified that have putative roles in host cell attachment and interaction. Proteins with emPAI scores >1.0 are shown in Table 2.

Proteins involved in redox homeostasis (thioredoxin, peroxiredoxin) were identified as highly expressed, which is unsurprising given the oxidative stresses imposed by the haem-rich intraerythrocytic environment. Heat shock proteins (HSPs) 70 and 90 were abundant in all genotypes of *T. orientalis* (Table 2). HSPs also play an important role in parasite homeostasis by maintaining the integrity of protein folding under thermal stress. This is thought to be particularly critical in apicomplexans that have lifecycle stages in both poikilothermic and homeothermic hosts, such as *Theileria, Babesia* and *Plasmodium* spp. In *P. falciparum*, HSPs are also involved in export of parasite proteins to the erythrocyte surface and regulation of pathogenesis, and consequently both HSP 70 and HSP 90 are major targets for antimalarial compounds [25]. Novobiocin has also been shown to inhibit *Babesia caballi* and *Theileria equi* in in vitro culture by targeting HSP 90 [26] suggesting that HSPs may also be suitable therapeutic targets for *T. orientalis*.

**Table 2:** Most abundant (emPAI > 1.0) aqueous phase proteins by *T. orientalis* genotype.

Genotype	Protein Name	Accession	emPAI	Function	Reference
Ikeda	ToLocg 1 protein	Q75R34	52.06	Unknown	[27]
	Uncharacterised protein	J4DPH8	10.83	Unknown	[23]
	Uncharacterised protein	J4CCC5	5.06	Unknown	[23]
	Uncharacterised protein	J7M4S6	4.93	Unknown	[23]
	Uncharacterised protein	J4C2Q4	3.36	Unknown	[23]
	Uncharacterised protein	J4C9B1	3.06	Unknown	[23]
	Uncharacterised protein	J4D7C0	1.88	Unknown	[23]
	Actin	S7VNC9	1.67	Cellular organisation and transport	[23]
	Elongation factor 1 alpha	J4D5 × 2	1.46	Translation	[23]
	Heat shock protein 70	J4D700	1.35	Protein processing and turnover	[25]
	Microneme-rhoptry related protein	D0FY42	1.33	Host cell interactions	[28]
	Heat shock protein 90	J4CCT6	1.29	Protein processing and turnover	[26]
	Peptidyl-prolyl cis-trans isomerase	J7MC36	1.29	Homeostasis	[27]
	Uncharacterised protein	J4DNX9	1.24	Unknown	[23]
	Nucleoside diphosphate kinase	J4C3B5	1.11	Metabolism—nucleotide synthesis	[29]
	Uncharacterised protein	J4C3S5	1.05	Unknown	[23]
	Deoxyuridine 5’-triphosphate nucleotidohydrolase	J4D5D2	1.04	Metabolism—nucleotide synthesis	[30]
	Thioredoxin	J7MGV8	1.02	Redox homeostasis	[31]
Buffeli	Elongation factor 1-alpha	L1LF41	1.99	Translation	[22]
	Heat shock protein 70	J4D700	1.81	Protein processing and turnover	[25]
	Glyceraldehyde 3-phosphate dehydrogenase	J4C4H6	1.59	Metabolism—glycolysis	[32]
	Uncharacterised protein	J4C2Q4	1.67	Unknown	[22]
	Uncharacterised protein	J4CCC5	1.46	Unknown	[22]
	Heat shock protein 90	J4CCT6	1.19	Protein processing and turnover	[26]
	GMP synthase	J4C7N6	1.09	Metabolism—nucleotide synthesis	[33]
	Deoxyuridine 5’-triphosphate nucleotidohydrolase	J4D5D2	1.04	Metabolism—nucleotide synthesis	[30]
	Peroxiredoxin	J7MCG4	1.04	Redox homeostasis	[34]
Chitose	Actin	S7VNC9	8.54	Cellular organisation and transport	[22]
	Beta tubulin	110644985	1.07	Cell division	[22]
	Heat shock protein 90	J4CCT6	1.04	Protein processing and turnover	[26]

Uncharacterised proteins were amongst the most abundant proteins in the aqueous phase fractions. Interestingly, in the pathogenic genotype, *T. orientalis* Ikeda, ToLocg 1 (*Theileria orientalis* low copy number gene 1) protein was the most abundant protein identified and was approximately 5× more abundant than the next most abundant protein. The function of this protein is unknown but homologs of ToLocg 1 are present in *Theileria parva* and *Theileria uilenbergi*, with the homlog in *T. uilenbergi* being used for ELISA development due to the fact that it is immunodominant in this species [27]. ToLocg 1 was also relatively abundant, although much less so (emPAI = 0.55) in the *T. orientalis* Buffeli aqueous phase. This protein was not identified in the Chitose genotype, and the gene encoding ToLocg 1 is absent from the Chitose genome [22]. Further investigation of the uncharacterised proteins revealed that the top 7 most abundant aqueous phase proteins in *T. orientalis* Ikeda (including ToLocg 1), belong to the same orthogroup as defined by Orthofinder [22]. Further searches with the EggNOG database v5.0.0 reveal that these seven proteins plus one additional (J4DNX9) match to two EggNOG orthologous groups (ENOG503KE5M and ENOG503KDN3; Figure S3). Both EggNOG groups are specific to *Theileria* spp., and higher in copy number compared to *T. parva* and *T. annulata*. All eight proteins have predicted signal peptides or transmembrane domains (Table S3). Only two of these proteins (J4CCC5 and J4C2Q4) were found to be expressed in Buffeli piroplasms and none were expressed in the Chitose genotype. Furthermore, the *T. orientalis* Chitose genome only contains genes for J4CCC5 and J4C2Q4, and a truncated gene for J4C9B1 (truncated by ~45%) but completely lacks the remaining five homologs. The difference in the presence and expression of these genes between the pathogenic and apathogenic genotypes is striking and the respective functions of members of this ortholog group is worthy of further investigation.

The most abundant proteins in the detergent phases of each *T. orientalis* genotype were proteins involved in interactions with host cells (Table 3). The major piroplasm surface protein (MPSP) and another well-defined piroplasm membrane protein, P23, dominated the detergent fraction of all three genotypes. These proteins are known to be immunodominant in piroplasm phase of *T. orientalis* [35,36] but are also expressed in other lifecycle stages [37]. In *T. orientalis* Ikeda, a variant of the protein tocp1, a cysteine protease homolog, was also identified, as was a sodium/glutamate symporter which is believed to be involved in fuelling the TCA cycle (see Section 3.4.1). ADP ribosylation factor (Arf) was also relatively abundant in *T. orientalis* Ikeda. Arf proteins are GTPases important in intracellular signalling events and in *T. gondii* mediate the release of effector molecules from dense granules [38]. *Theileria* spp. contain microspheres, equivalent to the dense granules of *T. gondii,* during the sporozoite phase but not the schizont phase [39]. It is unclear whether these are present during the piroplasm phase [40].

### 3.4. Metabolic Pathways

#### 3.4.1. Glycolysis and the Citric Acid (TCA) Cycle

**Figure 2:**
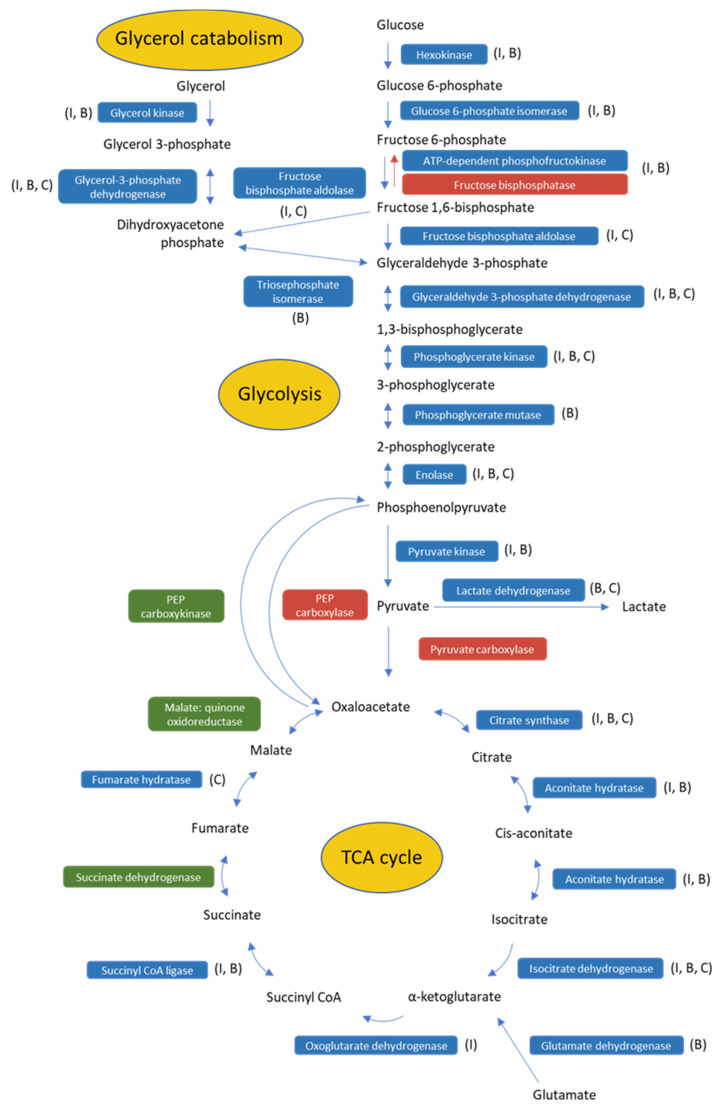
Metabolic pathways of glycolysis, glycerol catabolism and the TCA cycle with enzymes identified in *T. orientalis* piroplasms marked in blue, enzymes not identified in the piroplasm proteome in green and enzymes missing from both the piroplasm proteomes and the *T. orientalis* genomes in red. Genotypes in which each enzyme was identified are shown in brackets (I = Ikeda, B = Buffeli, C = Chitose).

Across all genotypes of *T. orientalis,* all enzymes involved in glycolysis were identified (Figure 2). Based on the presence of the substrate determining “GGDG” motif (Supplementary Figure S2), *T. orientalis* appears to use ATP-dependent phosphofructokinase (ATP-PFK) for the committed step of glycolysis which converts fructose-6-phosphate to fructose-1,6-bisphosphate, while in several other apicomplexans, pyrophosphate-dependent PFK (PPi-PFK) performs this function [32,46]. One key difference between these enzymes is that PPi-PFK can also catalyse this reaction in the reverse (gluconeogenic) direction. All *Theileria* spp. sequenced to date [24], along with some other apicomplexans such as *Plasmodium* spp. [47] lack key enzymes involved in gluconeogenesis, instead relying on the host for certain sugars. *T. orientalis* also appears to lack these enzymes including fructose bisphosphatase which catalyses the reverse reaction to ATP-dependent PFK, glucose-6-phosphatase, pyruvate carboxylase and phosphoenolpyruvate carboxylase, none of which were detected in this study and genes encoding these enzymes are also absent in the *T. orientalis* genome (Figure 2). Interestingly, enzymes involved in glycerol metabolism (glycerol kinase and glycerol-3-phosphate dehydrogenase) were also expressed in *T. orientalis* and were also relatively abundant despite being undetected in the *T. parva* sporozoite proteome [32] and detected at only very low levels in the *T. annulata* schizont proteome [24]. Glycerol may enter the glycolytic pathway or may be involved in glycerolipid synthesis. Indeed, diacylglycerol kinase (DGK) was also relatively abundant in *T. orientalis* Ikeda. DGK is involved in the production of phosphatidic acid, an important molecule in exocytosis.

Glycolysis is the major metabolic pathway used by the erythrocyte stages of a number of *Plasmodium* spp. [48] and this may also be true for *T. orientalis*. While most of the enzymes involved in the TCA cycle were identified as expressed in this study (Figure 2), their relative abundance was generally lower than seen for the glycolytic enzymes (Table S2). The lack of a mitochondrial pyruvate dehydrogenase complex in a number of apicomplexans [49] including *Theileria* spp. suggests that these pathways are not linked via the classical route involving acetyl CoA [44]. It has been suggested that glutamate may supplement the TCA cycle in *T. parva* [44] and *T. annulata* [24] and this may also be the case in *T. orientalis* piroplasms (Figure 2), supported by the relative abundance of a membrane-associated sodium/glutamate transporter in *T. orientalis* Ikeda (Table 3).

#### 3.4.2. Purine Metabolism

Apicomplexans, including *Theileria* spp., lack the ability to synthesise purine rings de novo and these are instead salvaged from the host. Because *Theileria* spp. do not reside intracellularly within a parasitophorous vacuole (this is degraded rapidly following cell entry [50]), such metabolites are readily available [51]. Based on genome sequences, *Theileria* spp. contain a limited number of enzymes that are involved in interconversion of purines and some of these were identified as expressed in the piroplasm phase of *T. orientalis*.

Adenylosuccinate lyase and adenylosuccinate synthetase, involved in the conversion of inosine monophosphate (IMP) to adenosine monophosphate (AMP), were both identified in the Ikeda genotype, as were IMP dehydrogenase and guanosine monophosphate (GMP) synthase, which are involved in the conversion of IMP to GMP.

#### 3.4.3. Amino Acid Metabolism

Apicomplexans also rely on the host for uptake of amino acids and a range of integral membrane proteins known as the Apicomplexan Amino acid Transporters (ApiATs) have been identified in *Toxoplasma* and *Plasmodium* spp. that are integral to the plasma membrane [51]. Based on amino acid homology there are 9 ApiATs in *T. orientalis* genome (J4CDF9, J4DPP7, J4C3V0, J4C3H6, J4D7Y9, J4DPC3, J4DNH3, J4D773, J4D8Y0) each with between 10 and 12 predicted transmembrane domains. Interestingly, none of these genes were detected in any *T. orientalis* genotypes in this study. In *Toxoplasma gondii*, expression of many of the ApiATs during the tachyzoite phase was limited or not detectable, suggesting that expression of these transporters might be specific to particular life cycle stages [52]. Ultrastructural studies show that *Theileria* spp. contain food vacuoles formed by endocytosis of host cell cytoplasm [53]. In the blood stage of *Plasmodium* spp., the food vacuole contains large quantities of haemoglobin which are broken down to provide the majority of amino acids for replication [54] and this may also be the case for the piroplasm phase of *Theileria* parasites. The Tocp1 protein is identified as relatively abundant in the *T. orientalis* Ikeda membrane fraction (Table 3), a cysteine protease-like protein with known haemoglobin-binding activity [45]. Furthermore, the papain family cysteine proteases (falcipains), falcilysin and aminopeptidases have all been implicated in the breakdown of haemoglobin in *Plasmodium* spp. [54,55,56,57,58], and all three types of proteases were found to be expressed in *T. orientalis* piroplasms in this study (Table 4). Falcilysin is being investigated as a potential drug target for *Plasmodium* as this metalloprotease is essential for viability of the blood stage of the parasite [59]; therefore, this may also be worth investigating as a chemotherapeutic target for *Theileria* spp.

*T. orientalis* also possesses a number of enzymes for the interconversion of amino acids including serine hydroxymethyltransferase (catalysing the interconversion of serine and glycine) and aspartate aminotransferase (catalyses conversion of aspartate to glutamate) and adenosylhomocysteinase (involved in methionine metabolism). A number of these enzymes have also been targeted for chemotherapeutic development in *Plasmodium* [61,62] and could be of utility in development of drugs for *Theileria* given the similar reliance of these parasites on a very limited repertoire of enzymes for amino acid metabolism.

### 3.5. Proteins Involved in Host Cell Interactions

A number of proteins were identified that are known to be involved in host cell adherence or other interactions, either in *Theileria* spp. or in other apicomplexans. These, along with other potential virulence factors, are listed in Table 5. Peptidyl prolyl cis/trans isomerases were detected in aqueous phases of all three *T. orientalis* genotypes. These proteins facilitate protein folding by isomerisation of proline residues. One enzyme from this family (PIN1) is known to be involved in induction of a cancer-like state in the transforming theilerias [63]. While the *T. orientalis* homolog of this protein lacks the signal peptide found in the transforming theilerias, buparvaquone interacts directly with the *T. annulata* PIN1, inhibiting the isomerase activity of this enzyme. Buparvaquone is also effective against *Theileria orientalis* infections, but it is unclear whether this is due to activity against the PIN1 orthologue as this drug also targets cytochrome b [64].

A homolog of *Plasmodium* hemolysin III was identified in the detergent phase of the Ikeda genotype. In *P. falciparum,* hemolysin III is a pore-forming protein expressed during the blood phase of the parasite. This protein is capable of erythrocyte lysis which may contribute to malaria-associated anaemia [65]. As anaemia is the major pathogenic effect of *T. orientalis* infection in cattle, further functional characterisation of this protein in *Theileria* spp. may be warranted.

A membrane attack complex (MAC)/perforin domain protein was identified in the Chitose genotype of *T. orientalis*. Proteins containing these domains are widespread in both prokaryotes and eukaryotes and they are responsible for forming pores in membranes of target cells. In apicomplexans, these proteins play important roles in the progression of the lifecycle, with species reliant on arthropod transmission (such as *Plasmodium*, *Babesia* and *Theileria* spp.) having a larger number of MAC/perforin domain proteins due to the need to traverse a wide variety of vertebrate and invertebrate host cell types [66]. Seven MAC/perforin domain proteins have been identified in *T. annulata*, with the *T. orientalis* protein most closely related to TA11680; however, there is little information about the cells targeted by these proteins. Expression of the *T. orientalis* MAC/perforin protein in the piroplasm phase of the lifecycle suggests that this homolog is most likely involved in traversing erythrocyte membranes.

Glycosylphosphatidylinositol (GPI) anchors are the most common carbohydrate modification in apicomplexan parasites and are critical for interactions with host cells. Many GPI anchored surface proteins have been identified in *Toxoplasma*, *Plasmodium* and *Cryptosporidium* and in the former two organisms the GPI biosynthetic pathway is essential for parasite survival [67]. GPI-anchored proteins also tend to be immunodominant [67,70]. For these reasons, GPI-anchored proteins are common targets for development of apicomplexan vaccines and chemotherapeutics [71,72,73]. A phosphatidylinositol glycan Q (PIG Q) homolog was identified in the detergent phase of the *T. orientalis* Ikeda genotype. PIG Q initiates the GPI biosynthetic pathway, suggesting that the GPI anchors are important in the erythrocyte phase of the *T. orientalis* lifecycle. Identification of GPI anchored proteins may assist in developing therapeutics for this species.

One such protein that is predicted to be GPI-anchored in *T. parva* is the 104 kDa microneme-rhoptry protein. Most apicomplexans produce micronemes, secretory organelles located at the apical end of sporozoites (and merozoites) that release proteins responsible for gliding motility and mediating cell entry [74]. However, *Theileria* sporozoites and merozoites are round, lacking an apical structure, are non-motile and lack discernible micronemes; although rhoptries (club-shaped secretory organelles usually associated with micronemes) are produced. P104 is the only known protein of the apical microneme-rhoptry complex found in *Theileria* spp. and is found on the sporozoite [75] and the schizont surface [76], where it is believed to interact with host cell microtubules. Sporozoite neutralising antisera react with p104 indicating that it might be a suitable vaccine candidate [73]. Here, we confirmed a prior study demonstrating that a homolog of p104 in *T. orientalis* (denoted ToMRP) [28] is also found in the red blood cell phase of the parasite, although interestingly this protein was sequestered in the aqueous phase rather than the detergent phase of the TX114 extraction, contrary to findings from proteomic analysis of the *T. annulata* schizont [24]. Prior studies of this protein indicate that it is expressed in the early and late erythrocytic stages and may be involved in invasion or egress from bovine red blood cells [28].

The MPSP and another piroplasm surface protein, P23, were amongst the most abundant detergent phase proteins identified in this study across all genotypes. These proteins are immunodominant [35,36] and the MPSP in particular has been the target for subunit vaccine development [41,42]. Furthermore, the MPSP is homologous to the *Theileria annulata* merozoite surface protein (Tams-1). Tams-1 is also highly expressed and immunodominant during the *T. annulata* merozoite phase and is considered a promising target for vaccine development [68]. Functional studies on these proteins are lacking but the *T. orientalis* MPSP has been implicated in binding to both heparin and bovine erythrocytes [43], suggesting that this protein may be involved in gaining erythrocyte entry. P23 also has a homolog in *T. annulata*, although the function has not yet been characterised. However, in *T. orientalis,* like the MPSP, P23 has been identified as a heparin-binding protein [35]. Indeed, as for *P. falciparum*, heparin has been shown to inhibit *T. orientalis* invasion of erythrocytes [77] and heparin analogues are considered promising chemotherapeutics for blood stage apicomplexan infections [78].

### 3.6. Future Directions

One of the limitations of this study was the relatively low number of proteins identified from each individual *T. orientalis* genotype, which precluded meaningful comparison of the protein expression pattern across pathogenic and non-pathogenic strains. A more in-depth proteomic characterisation between genotypes is warranted as this may improve understanding of the drivers of pathogenicity in the Ikeda vs. apathogenic types. Furthermore, characterisation of the expressed *T. orientalis* proteome during lifecycle phases occurring in the tick host, such as the sporozoite phase, would be useful direction for future research to elucidate differences in the biological processes occurring in the arthropod versus mammalian host.

## 4. Conclusions

This study provides the first global proteomic study of *T. orientalis* and the expressed proteins identified in the piroplasm stage of the lifecycle represent approximately one quarter of the total predicted proteome. While the number of expressed proteins identified did not allow for meaningful cross comparison of expression patterns between *T. orientalis* genotypes, this study provides clues to the metabolic and pathogenic processes used by the parasite during the intraerythrocytic lifecycle stage and thereby provides a basis for the development of chemotherapeutics or vaccines against key expressed proteins.

## Figures and Tables

**Table 1:** Aqueous and detergent phase proteins identified from individual genotypes of *T. orientalis*.

	*T. orientalis* Ikeda	*T. orientalis* Chitose	*T. orientalis* Buffeli	All Genotypes
Aqueous phase	272	179	378	829
Detergent phase	130	38	64	232
Total proteins *	402	217	442	1061

* Numbers reflect target proteins only. Host and contaminating proteins not included.

**Table 3:** Most abundant (emPAI > 0.5) detergent phase proteins by *T. orientalis* genotype.

Genotype	Protein Name	Accession	emPAI	Function	Reference
Ikeda	Major piroplasm surface protein	324021528	45.59	Host cell interaction	[41,42,43]
	Piroplasm surface protein P23	225320245	6.56	Host cell interaction	[35]
	Uncharacterised protein	J4C3E8	0.8	Unknown	[23]
	ADP-ribosylation factor	J4C807	0.58	Transcription, regulation and signalling	[38]
	Uncharacterised protein	J4DQ05	0.57	Unknown	[23]
	Sodium/glutamate transporter	J4DA94	0.52	Cellular organisation and transport	[24,44]
	Tocp1 variant	J4CDK5	0.52	Host cell interaction	[45]
Buffeli	Major piroplasm surface protein	747156609	8.86	Host cell interaction	[41,42,43]
	Piroplasm surface protein P23	225320237	3.38	Host cell interaction	[35]
	Genomic DNA chromosome 3 (putative porin)	Q4UBJ9	0.59	Cellular organisation and transport	[22]
Chitose	Major piroplasm surface protein	290883016	3.03	Host cell interaction	[41,42,43]
	Uncharacterised protein	J4CDD8	1.32	Unknown	[22]
	Piroplasm surface protein P23	B2LUF8	0.83	Host cell interaction	[35]

**Table 4:** Proteins expressed in *T. orientalis* piroplasms with potential roles in haemoglobin utilisation.

Accession	Annotation	Function	Reference
J4CDK5	Tocp 1	Binds haemoglobin	[28]
XP_001020177	Papain-family cysteine protease	Erythrocyte rupture, haemoglobin-hydrolysis	[58]
Q4N067	Cysteine proteinase	Vivapain-like, hydrolyses haemoglobin	[57]
J4C810	Falcilysin	Haemoglobin-degrading metalloprotease	[55]
J4DNF9	Aminopeptidase N	Degrades haemoglobin-derived peptides into amino acids	[60]

**Table 5:** Proteins involved in host cell interactions and their orthologs.

Accession	Annotation	Ortholog	Organism	Reference
J7MEL8	Peptidyl-prolyl cis-trans isomerase	TA18945	*T. annulata*	[63]
J7MF01 J7M8A7	Hemolysin III MAC/perforin domain protein	PBANKA-1319100 PY05180	*P. berghei* *P. yoellii*	[65] [66]
J4C901 D0FY42 J4DP87J4DP87	Phosphatidylinositol glycan Class Q Microneme-rhoptry related protein Major piroplasm surface protein (MPSP) Piroplasm membrane protein P23	YYG_01332 TpMuguga_04g00437 TA17050 TA13810	*P. vinckeii* *T. parva* *T. annulata* *T. annulata*	[67] [28] [68] [69]

## Data Availability

Data is contained within the article or Supplementary Materials. The data presented in this study are available in Table S2.

## Supplementary Materials

The following supporting information can be downloaded at: https://www.mdpi.com/article/10.3390/pathogens11101135/s1. Figure S1: aqueous and detergent phase protein fractions from TX-114 extraction of *T. orientalis*. Figure S2: Alignment of phosphofructokinase sequences from representative apicomplexans. Figure S3: Alignment of uncharacterised aqueous phase proteins found to be highly abundant in the T. orientalis Ikeda. Table S1: Number of *T. orientalis* proteins identified by functional group. Table S2: All proteins identified from the aqueous and detergent phases of *T. orientalis* Ikeda, Chitose and Buffeli, and their relative abundances. Table S3: Highly abundant uncharacterised protein orthologs identified in the *T. orientalis* Ikeda aqueous phase.
