# Supplementary material for: Characterisation of the Theileria orientalis Piroplasm Proteome across Three Common Genotypes
Source: Pathogens. 2022 Sep 30;11(10):1135. doi: 10.3390/pathogens11101135 (PMC9610513; doi:10.3390/pathogens11101135)
Supplement: Supplementary file 1 [file pathogens-11-01135-s001.zip › Figures.pdf]

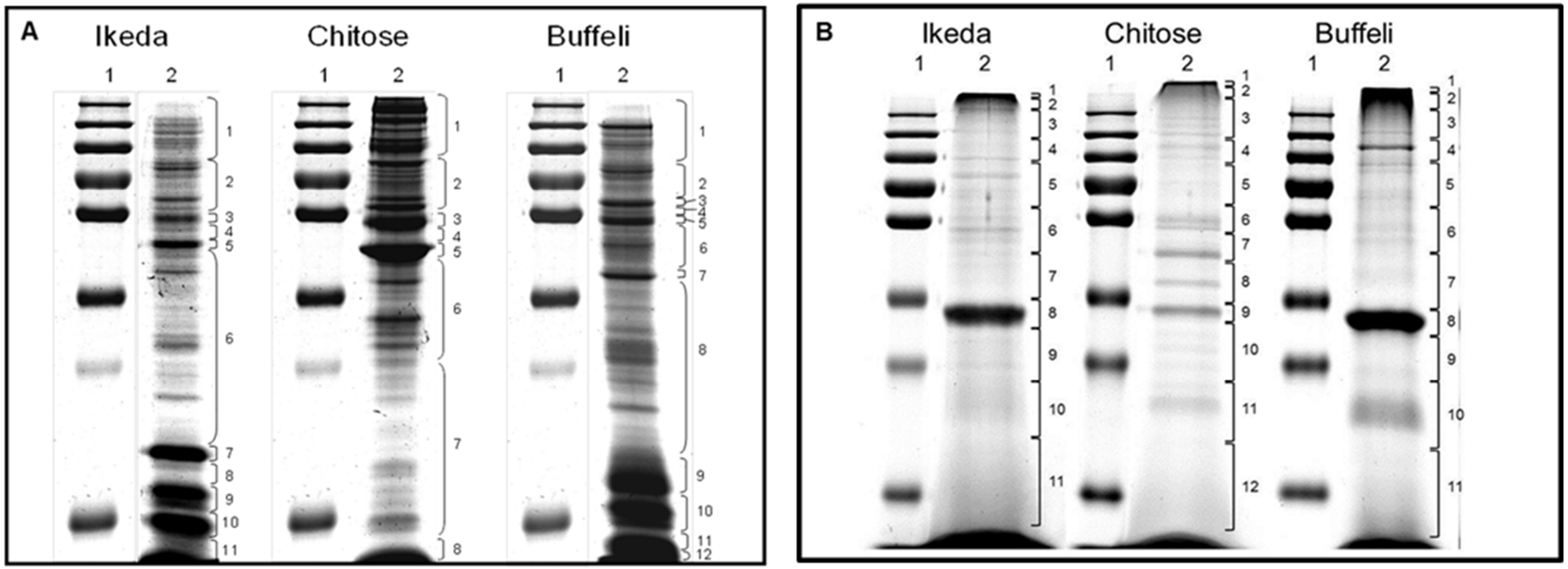

Figure S1. (**A**) Aqueous and (**B**) detergent phase protein fractions from TX-114 extractions of *T. orientalis* Ikeda, Chitose and Buffeli piroplasms were electophoresed on a 12% Tris-glycine one-dimensional SDS polyacrylamide gel. Lane 1 – PageRuler™ Prestained protein ladder (ThermoFisher Scientific); Lane 2 – *T. orientalis* protein sample. Bands from each protein sample were excised into 8-12 gel slices as indicated on the righthand side of the lane prior to processing for LC-MS/MS.

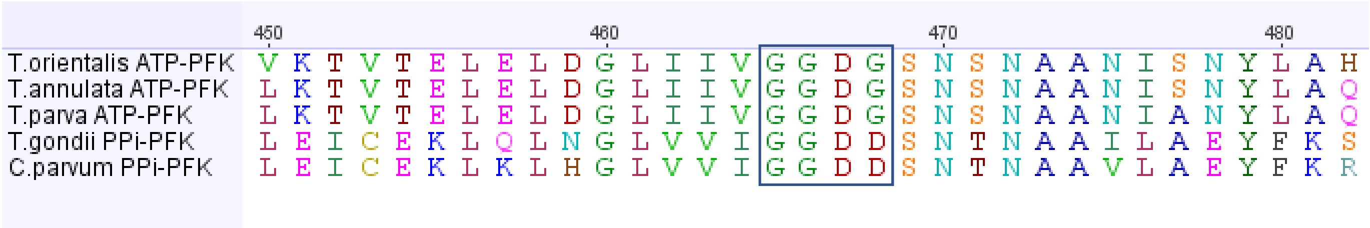

Figure S2. Alignment of phosphofructokinase sequences from representative apicomplexans showing the substrate-binding motif GGDG/D (boxed) which is associated with use of ATP or PPi for activity. *T. orientalis* PFK is predicted to be ATP-dependent.

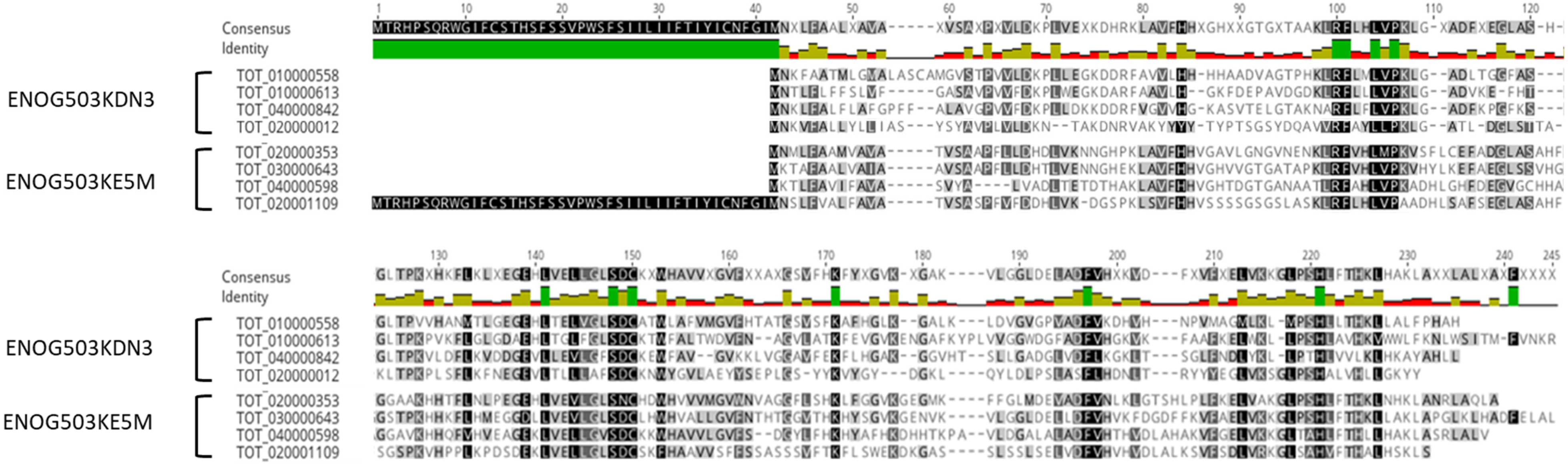

Figure S3. Alignment of uncharacterised aqueous phase proteins found to be highly abundant in the *T. orientalis* Ikeda piroplasm phase. These proteins are all orthologs and cluster into two phylogenetic groups according to EggNOG (ENOG503KDN3 and ENOG503KE5M).
